# A divergent *Anaplasma phagocytophilum* variant in an *Ixodes* tick from a migratory bird; Mediterranean basin

**DOI:** 10.1080/20008686.2020.1729653

**Published:** 2020-03-15

**Authors:** Tove Hoffman, Peter Wilhelmsson, Christos Barboutis, Thord Fransson, Thomas G.T. Jaenson, Per-Eric Lindgren, Friederike D. Von Loewenich, Åke Lundkvist, Björn Olsen, Erik Salaneck

**Affiliations:** a Department of Medical Biochemistry and Microbiology (IMBIM), Zoonosis Science Center, Uppsala University, Uppsala, Sweden; b Division of Inflammation and Infection, Department of Biomedical and Clinical Sciences, Linköping University, Linköping, Sweden; c Department of Clinical Microbiology, County Hospital Ryhov, Jönköping, Sweden; d Division of Medical Microbiology, Department of Clinical and Experimental Medicine, Linköping University, Linköping, Sweden; e Antikythira Bird Observatory, Hellenic Ornithological Society/Birdlife Greece, Athens, Greece; f Department of Environmental Research and Monitoring, Swedish Museum of Natural History, Stockholm, Sweden; g Department of Organismal Biology, Uppsala University, Uppsala, Sweden; h Department of Medical Microbiology and Hygiene, University of Mainz, Mainz, Germany; i Department of Medical Sciences, Zoonosis Science Center, Uppsala University, Uppsala, Sweden

**Keywords:** Bird migration, African-Western Palearctic region, *Hyalomma marginatum* s.l., *Ixodes*, *Anaplasma phagocytophilum*, 16s rRNA, *ankA*

## Abstract

*Anaplasma phagocytophilum* (AP) has vast geographical and host ranges and causes disease in humans and domesticated animals. We investigated the role of northward migratory birds in the dispersal of tick-borne AP in the African-Western Palearctic.

Ticks were collected from northward migratory birds trapped during spring migration of 2010 at two localities in the central Mediterranean Sea. AP DNA was detected by PCR (*gltA* and 16S rRNA) and variant determination was performed using *ankA* sequences.

In total, 358 ticks were collected. One of 19 ticks determined as *Ixodes* was confirmed positive for AP DNA. The tick was collected from a woodchat shrike (*Lanius senator senator*) trapped in Greece, and molecularly determined to belong to the *I. ricinus* complex and sharing highest (95%) 16S RNA sequence identity to *I. gibbosus*. The *ankA* AP sequence exhibited highest similarity to sequences from rodents and shrews (82%) and ruminants (80%). Phylogenetic analyses placed it convincingly outside other clades, suggesting that it represents a novel AP variant.

The divergent *Ixodes* species harboring a novel AP variant could either indicate an enzootic cycle involving co-evolution with birds, or dissemination from other regions by avian migration. None of the 331 *Hyalomma marginatum* sensu lato ticks, all immature stages, were positive for AP DNA, lending no evidence for the involvement of *Hyalomma* ticks transported by birds in the ecology of AP.

## Introduction

*Anaplasma phagocytophilum* (AP) (Family: *Anaplasmataceae*; Order: *Rickettsiales*) is an emerging, zoonotic, intracellular bacterium that may cause disease in humans and other mammals, mainly domesticated species such as cats [1], dogs [2], horses [3], cattle, sheep, and goats [4]. In ruminants the disease is called tick-borne fever and includes symptoms such as high fever, abortion, and a sudden drop in milk yield, resulting in economic losses for livestock owners. In humans, the disease is called human granulocytic anaplasmosis; here, the infection may range from asymptomatic to severe illness [5]. Underreporting of the disease is likely due to mild or asymptomatic infections in both humans and animals.

AP is present in Europe, North America, Asia, and Africa [6,7] and has a complex ecology, involving different vector species and mammalian host species. The bacterium also appears to have evolved into different strains or genetic variants, which may display different pathogenicities and host and/or vector preferences. In nature, AP is maintained through enzootic cycles between ticks and wild animals. Hard ticks (Ixodidae) of the *Ixodes ricinus* complex are the primary enzootic AP vectors and bridge vectors of AP to humans: *I. ricinus* (the common tick) in Western Eurasia; *I. persulcatus* (the taiga tick) in Eastern Eurasia; and *I. scapularis* (the deer tick) and *I. pacificus* (the western black-legged tick) in North America [7]. Their importance as enzootic and bridge vectors may differ between regions. In areas where the bridge vector may be absent, other tick species such as the nidicolous (non-questing, nest-dwelling) species *I. trianguliceps* may play a role in maintaining the enzootic cycles [8]. *Ixodes* species are known to transmit AP transstadially. Transovarial transmission has been investigated in *I. ricinus* but has not been demonstrated [9], possibly indicating that adult female ticks in the *I. ricinus* complex do not support vertical transfer to their offspring. This suggests that a vertebrate reservoir host, providing blood meals to susceptible vector competent ticks, is needed to maintain enzootic cycles of AP. In Europe, AP DNA has been detected in a wide range of wild animals, including roe deer (*Capreolus capreolus*), red deer (*Cervus elaphus*), wild boar (*Sus scrofa*), and small mammals [6]. However, the impact of AP infection in wild animals is unclear as well as their reservoir competence.

Geographical spread of AP may occur either via infected vertebrates or via infected ticks infesting hosts such as migratory birds [10,11]. The role of birds as reservoirs hosts of AP has been proposed [12] and suggested to be limited [13], but remains to be established. Every spring, millions of birds migrate from their wintering grounds in Africa, cross the Mediterranean Sea, and continue northward to their breeding grounds in the Palearctic region. This is one of the largest bird migration systems [14], in which Capri in Italy and Antikythira in Greece are two important stopover sites for birds arriving from Africa, after crossing the Sahara Desert and the Mediterranean Sea, and were therefore used as collection points in this investigation. Previous studies have shown that northward migrating birds, utilizing stopover sites in and near the Mediterranean Sea often are infested with ticks carrying zoonotic pathogens, both bacterial and viral [15–17]. In this study, the role of northward migratory birds in the dispersal of tick-borne AP in the African-Western Palearctic region was investigated using molecular detection methods.

## Materials and methods

### Bird trapping and tick collection

Ticks were collected from migratory birds trapped during the northward bound spring migration of 2010. The birds were trapped at two bird observatories on the islands of Antikythira (Greece; 35°51ʹN, 23°18ʹE) (March-May) and Capri (Italy; 40°33ʹN, 14°15ʹE) (April-May), using mist nets. Bird identification and tick collection were performed as previously described [16,18]. In brief, each bird was species identified and investigated for ticks around ears, neck, beak, and abdomen. Ticks were photographed and life stage and sex of adult ticks were recorded, and degree of blood engorgement estimated. Ticks were stored in RNAlater buffer (Qiagen, GmbH, Hilden, Germany) in −80°C. Ornithologists collected ticks while conducting the annual trapping and ringing of birds for other behavioral and ecological studies.

### Extraction of total nucleic acids and synthesis of cDNA

Extraction of total nucleic acids (NA) and synthesis of cDNA (enabling screening ticks also for RNA viruses) were performed as previously described [16]. Individual ticks were homogenized in 450 μL RLT buffer (Qiagen) supplemented with 1% β-mercaptoethanol (Sigma-Aldrich Sweden, Stockholm, Sweden), using a 5 mm stainless-steel bead (Qiagen) and a TissueLyser (Qiagen). Homogenization was performed for 2 minutes (min) at 25 Hz followed by 1 min at 25 Hz in room temperature. Homogenates were centrifuged for 3 min at 20,000 x g. Total NA was robot-extracted from 400 μL of supernatant, using the MagAttract RNA Tissue Mini M48 kit and the BioRobot M48 Workstation from Qiagen, according to the manufacturer´s instruction with the modification of not adding DNase to the RDD buffer. Total NA was eluted in 50 μL of RNase free water (VWR, PA, USA) and stored in −70°C. cDNA was synthesized using Illustra^TM^ Ready-to-Go RT PCR Beads kit (GE Healthcare, Amersham Place, UK), according to the instructions from the manufacturer. In brief, 20 μL of total NA was incubated with 10 μL (0.25 μg/μL) random hexamers (pd(N)6) for 5 min at 97°C. Beads (consisting of enzyme, nucleotides, and ions) were dissolved in 20 μL RNase-free water (VWR), mixed with the NA-primer solution, and incubated in a PTC-100 thermal cycler (M. J. Research, Inc., Waltham, MA) for 30 min at 42°C, followed by 5 min at 95°C.

### Tick species determination

Genus of the ticks was determined morphologically based on photographs taken in the field with a portable microscope and confirmed by sequence analysis of 10 randomly selected ticks. *Hyalomma* ticks were morphologically determined to the complex *H. marginatum*. For details see Wallménius et al. [16]. For specimen found positive for AP DNA or in cases with missing photographs, genus/species determination was performed molecularly targeting the mitochondrial 12S ribosomal RNA (rRNA) gene, the 16S rRNA gene, and the ribosomal internal transcriber spacer 2 (ITS2). See Table 1 for primer and probe sequences.

**Table 1.** Primers and probes used for analyses

Organism	Target gene	Name	Sequence (5´ → 3´)	Reference
Tick	12S rRNA	T1B	AAACTAGGATTAGATACCCT	[19]
		T2A	AATGAGAGCGACGGGCGATGT	
	16S rRNA	16s+1	CCGGTCTGAACTCAGATCAAG	[20]
		16S-1	GCTCAATGATTTTTTAAATTGCTG	
	ITS2	IXO-I2-F4	TCTCGTGGCGTTGATTTGC	[21]
		IXO-I2-R4	CTGACGGAAGGCTACGACG	
		Ipe-I2-P4	FAM -TGCGTGGAAAGAAAACGAG- BHQ1	
		Iri–I2-P4	VIC -TGCTCGAAGGAGAGAACGA- BHQ1	
AP	*gltA*	ApF	TTTTGGGCGCTGAATACGAT	[22]
		ApR	TCTCGAGGGAATGATCTAATAACGT	
		ApM	TGCCTGAACAAGTTATG	
	16S rRNA	16S-F5	AGTTTGATCATGGTTCAGA	[23]
		ANA-R4B	CGAACAACGCTTGC	
	*ankA*	SLO fo 1	GGGATRAGTGCRGTGCAGYAT	[24]
		SLO re 1	ACTGCRGCMGCTARAGGRCT	
		SLO fo 2	TTACGCTGTRRTRGCATRGAC	
		SLO re 2	AWRGWTCCSKYAGGAGYATTTA	

AP = *Anaplasma phagocytophilum*

The 12S and 16S PCR assays were performed as previously described with minor modifications [19,20], using a BioRad T100 (Bio-Rad Laboratories). Each 12S PCR reaction (25 µL) consisted of: 2.5 µL buffer (10x), 800 µM dNTPs (Invitrogen, Thermo Fisher Scientific), 3.5 mM MgCl_2_ (AB, Thermo Fisher Scientific), 1 µM of each primer (T1A/T2B, Invitrogen, Thermo Fisher Scientific), 0.5 U Platinum Taq (Invitrogen, Thermo Fisher Scientific), 2.5 µL template, and 9.4 µL sterile water (Thermo Fisher Scientific). Temperature profile: 95°C for 5 min; 5 cycles of 95°C for 15 seconds (s), 51°C for 30 s, and 68°C for 30 s; 25 cycles of 95°C for 15 s, 52°C for 30 s, and 70°C for 30 s; followed by a final elongation step at 72°C for 7 min. The amplicon size was 320 base pairs (bp). For the 16S PCR, each PCR reaction (25 µL) contained: 2.5 µL PCR Buffer (10x), 800 µM dNTP (Invitrogen, Thermo Fisher Scientific), 1.5 mM MgCl_2_ (AB, Thermo Fisher Scientific), 500 nM of each primer (16s+1/16s-1, TibMolBio, Berlin, Germany), 0.5 U Platinum Taq (Invitrogen, Thermo Fisher Scientific), 1.5 µL template, and 14.9 µL sterile water (Thermo Fisher Scientific). The thermal profile was: 94°C of 8 min; 2 cycles of 94°C for 1 min, 48°C for 1 min, 72°C for 2 min; 33 cycles of 94°C for 1 min, 54°C for 1 min, 72°C for 2 min; followed by a final elongation step at 72°C for 7 min. The final amplicon size was 460 bp.

The assay targeting the ITS2 was an *I. ricinus* or *I. persulcatus* species-specific duplex real-time (q) TaqMan™ PCR, performed as previously described [21]. The final qPCR reaction (20 µl) consisted of 10 µl Maxima® Probe qPCR Master Mix (2X) (Thermo Fisher Scientific), 200 nM of each primer (Invitrogen, Thermo Fisher Scientific), 150 nM of probe Iri–I2-P4 (Life technologies, Thermo Fisher Scientific), 100 nM of probe Ipe-I2-P4 (Life technologies, Thermo Fisher Scientific), 6.7 µl RNAse-free water (Life technologies, Thermo Fisher Scientific), and 2 µl template. Reactions were performed on a C1000^TM^ Thermal Cycler, CFX96^TM^ Real-Time PCR Detection System (Bio-Rad Laboratories, Inc., Hercules, CA) using the following temperature profile: 95°C for 5 min followed by 45 cycles of 95°C for 10 s and 60°C for 60 s.

### Detection of Anaplasma phagocytophilum *DNA*

Ticks were analyzed using a two-step TaqMan qPCR specific for a 64 bp segment of the *gltA* gene, encoding the enzyme citrate synthase, of AP as previously described [22]. The PCR reaction of 25 μL contained: 12.5 μL TaqMan Universal PCR Master Mix (2X, Applied Biosystems (AB), Foster City, CA, USA), 600 nM of each primer (Life Technologies, Thermo Fisher Scientific, Stockholm, Sweden), 150 nM minor groove binding (MGB®) probe (Life Technologies), 7.13 μL RNase-free water (VWR), and 2 μL template. The temperature profile was as follows: UNG treatment at 50°C for 180 s initial denaturation at 95°C for 10 s, followed by 40 cycles of 95°C for 15 s and 60°C for 60 s. The CFX96^TM^ Real-Time PCR Detection System from Bio-Rad Laboratories (Inc., Hercules, CA, USA) was used for the analyses. As a positive control and to quantify the number of *Anaplasma gltA* genes, a serial dilution of a synthetic plasmid containing the target sequence of the TaqMan qPCR assay was used. The plasmid contained the target sequence, spanning the nucleotides 304–420 of the AP *gltA* gene (Accession no.: AF304137), synthesized and cloned into pUC57 vector (Genscript USA Inc, NJ).

Subsequent confirmation analysis was performed on samples positive in the TaqMan qPCR assay by conventional PCR, using the first primer pair of the AP specific 16S PCR by Stuen et al. [23], producing an amplicon of 507 (bp) (Table 1). For variant determination, a segment of *ankA*, a gene encoding an ankyrin repeat protein presumably involved in host-specific adaptation [24], was amplified and sequenced as described [24]. A positive (AP-DNA) and negative control (sterile water) were included in the PCR analyses.

### Phylogenetic analyses

The 12S and 16S rRNA gene amplicons were treated with Illustra ExoProStar 1-step (GE Healthcare, Stockholm, Sweden) and sequenced at Macrogen (Amsterdam, the Netherlands). Obtained sequences were trimmed and assembled in the CLC Main Workbench 7 (Qiagen, Aarhus, Denmark) and aligned using the MAFFT algorithm (https://www.ebi.ac.uk/Tools/msa/mafft/) [25], with default settings. Reference sequences were retrieved from the NCBI GenBank database (https://www.ncbi.nlm.nih.gov/genbank/). Phylogenies were built in MEGA7 [26], using the Maximum Likelihood (ML) and Neighbor-Joining (NJ) algorithms. For the ML analyses the following substitution models combined with the models of gamma distribution (G) and invariable sites (I) were chosen, using model testing in MEGA7: General Time Reversible (GTR+G+I) (16S rRNA gene *Ixodes* alignment), Hasegawa-Kishino-Yano (HKY+G) (12S rRNA gene *Ixodes* alignment), and Kimura-2 parameter (K2P+G+I) (16S rRNA gene *Anaplasma* alignment). For the NJ analyses, the default choice the Maximum Composite Likelihood model was used. Gaps were treated using the complete deletion option. The bootstrap analysis was conducted with 1,000 replicates. The 12S and 16S rRNA gene tick sequences amplified in this study have been deposited in GenBank (Accession no.: MN263069, MN263068, MN252874).

The partial *ankA* sequence described here was compared to 398 *ankA* sequences published earlier [24,27]. Accession numbers are listed in table 4 (supplementary material). The program MEGA X version 10.0.5 was used for phylogenetic analyses [28]. *ankA* sequences were codon-aligned by ClustalW applying the PAM (Dayhoff) matrix. Tree construction was achieved by the ML method using the Tamura-Nei model and a gamma distribution (TN93+G) and the NJ method using the Jukes-Cantor (JC) model, with the complete deletion option. Bootstrap analysis was conducted with 1,000 replicates. Net average identities at the nucleotide level and net average similarities at the amino acid level between the different *ankA* gene clusters described previously [24] and the tick sequence found here were calculated using MEGA X. The *ankA* sequence amplified in this study has been deposited in GenBank (Accession no.: MN062926). NJ phylogenies are presented upon request.

## Results

### Study material

In 2010, a total of 7,354 birds were trapped and examined for ticks [16]. Three hundred fifty-eight (n = 358) ticks (Antikythira: n = 200; Capri: n = 158) were collected from 203 birds (Antikythira: n = 103; Capri: n = 100) of 27 species (Antikythira: n = 26; Capri: n = 11) (Table 2), mainly long-distance migrants foraging on the ground and in shrubs and trees. The tick infestation frequency was approximately 3.1% (203/7,354) with an average of 1.8 ticks per infected bird (358/203). The collected tick population was comprised of: 92.5% (331/358) *H. marginatum* sensu lato (s.l.); 5.3% (19/358) *Ixodes*; 0.3% (1/358) *Haemaphysalis*; 0.3% (1/358) *Rhipicephalus*; and 1.7% (6/358) was of unidentified origin. Regarding the life stage, 22.9% (82/358) were larvae, 64% (229/358) were nymphs, 0.8% (3/358) were adults, and 12.3% (44/358) were of unknown stage. Estimation of blood engorgement level was possible for 82% (294/358) of the collected ticks. Of these, 1% (3/294) were unfed, 17.7% (52/294) were partially fed, and 81.3% (239/294) were fully fed. See Table 3 for data concerning each specific tick genus. Missing data on tick genus, life stage, and level of engorgement was due to absence or poor quality of photographs and PCR amplicon or template.

**Table 2.** Bird species infested with ticks trapped on Antikythira (Greece) and Capri (Italy) during the spring migration (March to May) of 2010

		Tick life stage				
Bird species *(Latin name)*	No. of birds infested/ No. of birds examined (%)	No. of larvae	No. of nymphs	No. of adults	ND	No. of ticks
Great Reed Warbler	3/197 (1.5)	0	11	0	0	11
*(Acrocephalus arundinaceus)*						
Sedge Warbler	7/202 (3.5)	2	19	0	2	23
*(Acrocephalus schoenobaenus)*						
Reed Warbler	1/14 (7.1)	0	2	0	0	2
*(Acrocephalus scirpaceus)*						
Tree Pipit	5/201 (2.5)	0	5	0	0	5
*(Anthus trivialis)*						
European Nightjar	1/25 (4.0)	0	0	0	2	2
*(Caprimulgus europaeus)*						
European Greenfinch	1/21 (4.8)	0	0	0	4	4
*(Chloris chloris)*						
European Robin	6/81 (7.4)	7	5	0	0	12
*(Erithacus rubecula)*						
Collared Flycatcher	2/131 (1.5)	2	0	1*	0	3
*(Ficedula albicollis)*						
European Pied Flycatcher	26/981 (2.7)	21	16	0	2	39
*(Ficedula hypoleuca)*						
Icterine Warbler	2/184 (1.1)	2	0	0	1	3
*(Hippolais icterina)*						
Olivaceous Warbler	1/22 (4.5)	0	1	0	0	1
*(Iduna pallida)*						
Woodchat Shrike	11/91 (12.1)	1	13	0	11	25
*(Lanius senator)*						
Nightingale	7/202 (3.5)	10	10	0	0	20
*(Luscinia megarhynchos)*						
Yellow Wagtail	1/4 (25)	1	1	0	0	2
*(Motacilla flava)*						
Spotted Flycatcher	5/467 (1.1)	2	3	0	0	5
*(Muscicapa striata)*						
Northern Wheatear	6/49 (12.2)	1	18	0	0	19
*(Oenanthe oenanthe)*						
Eurasian Golden Oriole	6/147 (4.1)	3	4	0	0	7
*(Oriolus oriolus)*						
Common Redstart	19/207 (9.2)	4	19	0	8	31
*(Phoenicurus phoenicurus)*						
Eastern Bonelli’s Warbler	4/33 (12.1)	2	2	0	3	7
*(Phylloscopus orientalis)*						
Wood Warbler	14/696 (2.0)	8	4	1*	2	15
*(Phylloscopus sibilatrix)*						
Willow Warbler	1/274 (0.4)	0	0	1*	0	1
*(Phylloscopus trochilus)*						
Whinchat	36/731 (4.9)	9	52	0	0	61
*(Saxicola rubetra)*						
Eurasian Siskin	1/3 (33.3)	0	1	0	0	1
*(Spinus spinus)*						
Garden Warbler	7/1,186 (0.6)	5	1	0	1	7
*(Sylvia borin)*						
Common Whitethroat	26/382 (6.8)	2	34	0	1	37
*(Sylvia communis)*						
Song Thrush	2/16 (12.5)	0	6	0	7	13
*(Turdus philomelos)*						
Eurasian Hoopoe	2/10 (20)	0	2	0	0	2
*(Upupa epops)*						
*Total*	203/6,557 (3.1/89.2)	82	229	3	44	358
*Total no. of captured birds*	7,354					

ND = unidentified

* female tick

**Table 3.** Life stage and estimated level of blood engorgement of ticks infesting northward migratory birds trapped on Antikythira (Greece) and Capri (Italy) during the spring migration of 2010

		Life stage					Level of blood engorgement			
Tick genus	Total (%)	Larva (%)	Nymph (%)	Adult (%)	Female (%)	Unidentified (%)	Fully fed (%)	Partially fed (%)	Unfed (%)	Unidentified (%)
*Hyalomma*	331 (92.5)	74 (22.2)	221 (66.8)	0 (0)	0 (0)	35 (10.6)	229 (69.2)	45 (13.6)	2 (0.6)	55 (16.6)
*Ixodes*	19 (5.3)	7 (36.8)	6 (31.6)	3 (15.8)	3 (15.8)	3 (15.8)	8 (42.1)	7 (36.8)	1 (5.3)	3 (15.8)
*Haemaphysalis*	1 (0.3)	0 (0)	1 (100)	0 (0)	0 (0)	0 (0)	1 (100)	0 (0)	0 (0)	0 (0)
*Rhipicephalus*	1 (0.3)	1 (100)	1 (100)	0 (0)	0 (0)	0 (0)	1 (100)	0 (0)	0 (0)	0 (0)
Unidentified	6 (1.7)					6 (100)				6 (100)
**Total (%)**	358 (100)	82 (22.9)	229 (64)	3 (0.8)	3 (0.8)	44 (12.3)	239 (66.8)	52 (14.5)	3 (0.8)	64 (17.9)

### Presence of Anaplasma phagocytophilum DNA

AP DNA was detected in one (n = 1) out of 19 *Ixodes* ticks (5.3%). The *Ixodes* sp. tick was collected from a woodchat shrike (*Lanius senator senator*), trapped on the island of Antikythira in 2010. The copy number of the *gltA* gene in the positive tick was determined to be approximately 4 × 10^3^.

### Phylogenetic analyses

#### Phylogenetic analyses of tick species

**Figure 1.**
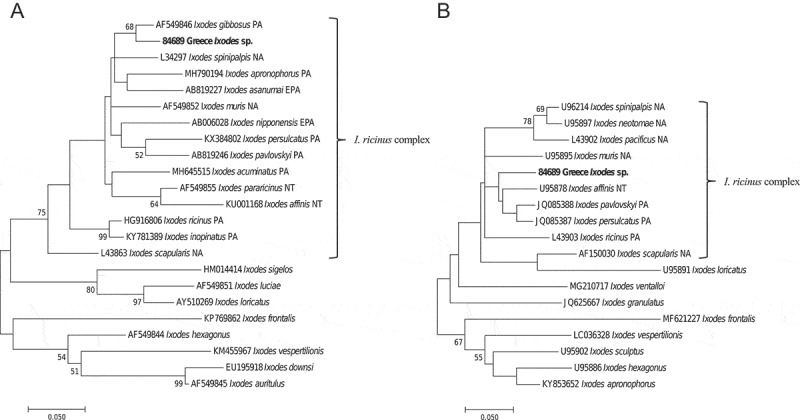
Evolutionary relationship of the *Anaplasma phagocytophilum* positive tick

Molecular species determination of the AP-positive tick was performed using three different molecular markers. No amplicons were generated when using the *ITS2* PCR [21], indicating that the investigated tick was neither *I. ricinus* nor *I. persulcatus*. In the *Ixodes* phylogenies, the 12S and 16S rRNA gene sequences from the positive tick (84689) grouped together with sequences from the *I. ricinus* complex (Figure 1A-B), such as *I. ricinus, I. persulcatus, I. scapularis*, and *I. gibbosus* [29]. In the 16S phylogeny (Figure 1A), the tick sequence formed a clade (68% bootstrap support) with *I. gibbosus* (GenBank accession no.: AF549846). At the nucleotide level, the tick 16S rRNA sequence had an identity of 95% (328/345) to the sequence of *I. gibbosus*, including three gaps and 14 mismatching nucleotides.

Phylogenies of tick species within the genus *Ixodes*. The study sequence (in bold) appears together with tick species of the *I. ricinus* complex. **A**) Phylogenetic comparison of partial *Ixodes* 16S rRNA gene sequences inferred by using the Maximum Likelihood method, based on the General Time Reversible model (GTR+G+I). A total of 315 positions was in the final dataset. **B**) Phylogenetic comparison of partial *Ixodes* 12S rRNA gene sequences inferred by using the Maximum Likelihood method, based on the Hasegawa-Kishino-Yano model (HKY+G). No suitable 12S rRNA gene sequence of *I. gibbosus* was available in GenBank. A total of 316 positions was in the final dataset. Branch lengths are measured in the number of substitutions per site and bootstrap values of more than 50% are presented. Evolutionary analyses were conducted in MEGA7 [26]. Accession numbers and geographical distribution of tick species within the *I. ricinus* complex are presented in the trees. PA = Palearctic region; EPA = Eastern Palearctic region; NA = Nearctic region; and NT = Neotropical region.

#### *Phylogenetic analyses and variant determination of* Anaplasma phagocytophilum

**Figure 2.**
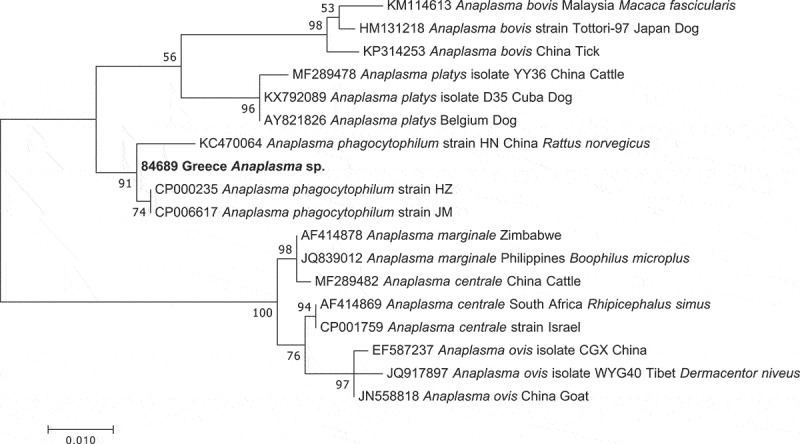
Evolutionary relationship of the *Anaplasma* 16S rRNA gene sequence from the tick infesting a woodchat shrike (*Lanius senator senator*). Phylogenetic tree based on partial *Anaplasma* 16S rRNA gene sequences. The study sequence (in bold) appears within the *A. phagocytophilum* clade. The phylogeny was inferred using the Maximum Likelihood method, based on the Kimura 2-parameter model (K2P+G+I) and the complete deletion option. The percentage of replicate trees in which the associated taxa clustered together in the bootstrap test (1,000 replicates) is shown next to the branches. Branch lengths represent the number of substitutions per site. The final data set contained 478 positions. Evolutionary analysis was conducted in MEGA7 [26]. Accession numbers and source information are presented in the tree

**Figure 3.**
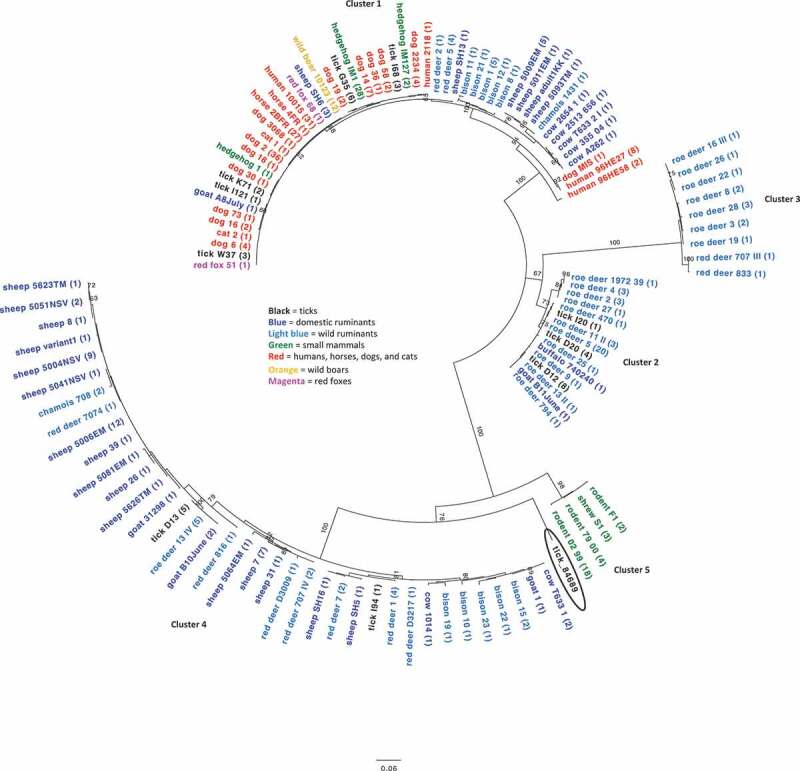
Phylogenetic tree derived from partial *Anaplasma phagocytophilum ankA* gene sequences

Phylogenetic analyses of partial *Anaplasma* spp. 16S rRNA gene sequences (Figure 2), showed that the 16S rRNA gene sequence from the positive tick (84689) clustered with sequences of AP. The novel *ankA* sequence was found to be an outgroup of the *ankA* cluster 4 (consisting of sequences from domestic and wild ruminants) (Figure 3), and shared a nucleotide identity of 80% and 82% with clusters 4 and 5 (consisting of sequences from rodents and shrews), respectively. At the amino acid level, the similarity was 62% and 59% to cluster 4 and 5, respectively

The study sequence (circled), retrieved from an *Ixodes* species tick, appears on a branch between cluster 4 and 5. Sequence names are colored by host groups and identical sequences are displayed only once per species. The number in parenthesis indicates the frequency with which the respective sequence was found. Tree construction was achieved by the Maximum Likelihood method, using the Tamura-Nei model (TN93 + G) with the complete deletion option. Bootstrap values less than 60% are not shown. The scale bar indicates the number of nucleotide substitutions per site. The final data set contained 500 positions. Evolutionary analysis was conducted in MEGA X [28]. Accession numbers are listed in table 4 (supplementary material).

## Discussion

In the present study, the role of northward spring migratory birds in the dispersal of tick-borne AP in the African-Western Palearctic region was investigated

One out of 358 (0.28%) analyzed ticks, was determined to contain AP DNA (Figure 2). Phylogenetic analyses placed the AP DNA containing tick with tick species of the *I. ricinus* complex (Figure 1), a group of closely related tick species that includes the main vector of AP in Europe (*I. ricinus*) [7,30]. The negative results of the ITS2 assay strongly suggest that this tick is neither *I. ricinus* nor *I.* *persulcatus*. Furthermore it appeared within a clade (68% bootstrap support) together with *I. gibbosus* (Figure 1A), a tick species found in the drier and warmer parts of the Mediterranean basin, including Greece [31]. Reports of *I. gibbosus* from Africa are absent at present, but the extent of investigations in the area is not evident. The observed percentage of dis-identity (5%) between the study tick 16S rRNA gene sequence and that of *I. gibbosus* (AF549846, originating from a tick collected in Turkey) indicates that they could represent different tick species or subspecies, or reflect the different geographic origin of the specimens, which has been discussed for the 16S rRNA gene by Mangold and co-authors [20]. Unfortunately, lack of material prevented further investigation of this particular question.

The *Ixodes* tick was collected in 2010 from a woodchat shrike trapped on the island of Antikythira, a small and remote island situated north-west of Crete in the Aegean Sea in southern Greece. The woodchat shrike is a medium sized long-distance migrating passerine that winters in sub-Saharan Africa and breeds in many areas around the Mediterranean Sea and in the Middle East [32]. Antikythira is used only as a stopover site by woodchat shrikes [33] and is one of the first stops near the European continent during the spring migration. Besides harboring a large number of migrating birds, Antikythira also harbors a relatively large population of free-roaming feral goats (*Capra aegagrus hircus*) [34], rodents (e.g. black rats (*Rattus rattus*), and house mice (*Mus musculus*)) (C. Barboutis, personal communication), which could serve as hosts for ixodid ticks. The mere presence of bacterial DNA in a tick is not sufficient to determine bacterial infectivity and that the results could alternatively reflect either a host bacteremia or carriership of the pathogen by the tick. Depending on the life stage of the *Ixodes* tick (unknown due to absence of a photograph) and in the potential lack of transovarial transmission, the tick may have acquired AP from its avian host, by potential co-feeding, or from a previous infective mammalian or avian host through transstadial transmission.

The degree of susceptibility of a particular host species to AP as well as zoonotic potential seems to be related to particular genetic variants of AP. Analyses of the *ankA* and *groEL* (heat shock protein) genes in AP have revealed clusters according to host species origin [24,35–37]. Variants of AP found in humans and domestic animals have been found to cluster with sequences from ticks and wildlife, indicating a zoonotic cycle, while variants of AP found in rodents and shrews as well as in birds and bird-derived ticks have been found to differ, possibly indicating divergent enzootic cycles [24,35,36]. The *ankA* sequence in this study (Figure 3), exhibited the highest nucleotide identity to cluster 5 sequences of rodents and shrews (82%) and cluster 4 from ruminants (80%), but appeared convincingly outside both of these clades. We therefore suggest that this is a bird-specific AP variant that could represent an enzootic cycle with birds as hosts. However, the variant could also represent a novel enzootic cycle involving local island biogeographic conditions (geographical isolation) or influx of an infected tick from another area by avian migration. Which area this could be is unclear, but migratory patterns of the woodchat shrike primarily suggest Northern or sub-Saharan Africa, warranting further investigation of birds, bird associated ticks, and potential mammalian hosts there as well as in Greece and neighboring areas. Since the *ankA* gene might be subjected to recombination [37], multiple molecular markers should also be utilized in these studies. AP has seldomly been detected in birds and/or avian-derived ticks and the role of birds, including the potential role of the woodchat shrike, as reservoir competent hosts of zoonotic AP variants should therefore be addressed. This should include investigations of infection susceptibility (development of bacteremia) and level and duration of infectivity in birds in order to further elucidate the transmission cycles of AP.

AP DNA has been detected in several other tick species, including *Dermacentor marginatus, Rhipicephalus sanguineus, H. lusitanicum*, and *H. marginatum* [38,39]. Their role in the ecology and epidemiology of AP and their vector competence are however unclear. A majority of the collected ticks (92.5%) in this study belonged to the *H. marginatum* complex, and likely represented the species *H. marginatum* and *H. rufipes* [40]. AP DNA has been detected in adult *H. marginatum* ticks collected in France, Israel, and Africa [38,41,42]. The distribution of AP in Africa is not well studied and reports of clinical human cases on the African continent are to our knowledge absent. Eighty-seven percent (87%) of the investigated ticks were larvae and nymphs. Immature *H. marginatum* and *H. rufipes* commonly parasitize wild birds and molt from larva to nymph on the same individual host [40]. This enables long distance dispersal on migrating birds. The *Hyalomma* specimens investigated here, likely originated from sub-Saharan or North Africa. We could not detect AP DNA in any of the *Hyalomma* ticks. This may reflect that immature *H. marginatum* s.l. ticks and migratory bird hosts do not play an important role in the ecology of AP, at least not in the investigated regions.

## Conclusion

We report the detection of a divergent AP variant in a potentially novel *Ixodes* tick species, sub-species, or variant within the *I. ricinus* complex, but not *Ixodes ricinus*, the primary AP vector in the region. Since this tick was collected from a bird, this could indicate an avian associated enzootic cycle, warranting further investigation. Furthermore, our data does not provide evidence for immature *H. marginatum* s.l. ticks and birds having any major role in the ecology and northward dispersal of AP in the African-Western Palearctic region.

## Supplementary Material

Supplemental Material
