# Supplementary material for: A divergent Anaplasma phagocytophilum variant in an Ixodes tick from a migratory bird; Mediterranean basin
Source: Infect Ecol Epidemiol. 2020 Mar 15;10(1):1729653. doi: 10.1080/20008686.2020.1729653 (PMC7144310; doi:10.1080/20008686.2020.1729653)
Supplement: Supplemental Material [file ZIEE_A_1729653_SM0015.docx]

**Table 4.** GenBank accession numbers and denoted clusters for *ankA* sequences presented in Figure 3.

| **Sequence name** | **Accession number** | ***ankA* cluster** |
| --- | --- | --- |
| bison 1 | GU236733 | 1 |
| bison 10 | GU236737 | 4 |
| bison 11 | GU236738 | 1 |
| bison 12 | GU236739 | 1 |
| bison 15 | GU236740 | 4 |
| bison 19 | GU236743 | 4 |
| bison 21 | GU236745 | 1 |
| bison 22 | GU236746 | 4 |
| bison 23 | GU236747 | 4 |
| bison 8 | GU236735 | 1 |
| buffalo 740240 | MH987749 | 2 |
| cat 1 | GU236864 | 1 |
| cat 2 | FJ515309 | 1 |
| chamois 1431 | JQ347590 | 1 |
| chamois 708 | JQ347589 | 4 |
| cow 1014 | GU236732 | 4 |
| cow 2513 656 | GU236729 | 1 |
| cow 2654 1 | GU236730 | 1 |
| cow 355 04 | GU236731 | 1 |
| cow A262 | KC740482 | 1 |
| cow T633 1 | KC776917 | 4 |
| cow T633 2 I | KC776919 | 1 |
| dog 14 | GU236824 | 1 |
| dog 16 | GU236826 | 1 |
| dog 18 | GU236828 | 1 |
| dog 19 | GU236829 | 1 |
| dog 2 | GU236817 | 1 |
| dog 2234 | GU236814 | 1 |
| dog 30 | GU236838 | 1 |
| dog 3068 | GU236816 | 1 |
| dog 36 | GU236843 | 1 |
| dog 58 | GU236854 | 1 |
| dog 6 | GU236819 | 1 |
| dog 73 | KF242674 | 1 |
| dog MIS | KC740481 | 1 |
| goat 1 | KF242660 | 4 |
| goat 31298 | MH997028 | 4 |
| goat A8July | MH987720 | 1 |
| goat B10June | MH997029 | 4 |
| goat B11June | MH987751 | 2 |
| hedgehog 1 | KF242693 | 1 |
| hedgehog IM1 | KF242694 | 1 |
| hedgehog IM127 | KF242705 | 1 |
| horse 2BFR | GU236855 | 1 |
| horse 4FR | GU236857 | 1 |
| human 10015 | JQ347547 | 1 |
| human 2118 | GU236802 | 1 |
| human 96HE27 | KC740478 | 1 |
| human 96HE58 | GU236808 | 1 |
| red deer 1 | GU236724 | 4 |
| red deer 2 | GU236725 | 1 |
| red deer 5 | GU236727 | 1 |
| red deer 7 | GU236728 | 4 |
| red deer 707 III | GU236720 | 3 |
| red deer 707 IV | GU236721 | 4 |
| red deer 7074 | GQ428332 | 4 |
| red deer 816 | JQ347570 | 4 |
| red deer 833 | JQ347571 | 3 |
| red deer D3009 | JQ347573 | 4 |
| red deer D3217 | JQ347587 | 4 |
| red fox 51 | KC763008 | 1 |
| red fox 68 | KC763009 | 1 |
| rodent 02 99 | KC740451 | 5 |
| rodent 79 00 | KC740472 | 5 |
| rodent F1 | KC740470 | 5 |
| roe deer 11 II | GU236874 | 2 |
| roe deer 13 II | GU236877 | 2 |
| roe deer 13 IV | GU236878 | 4 |
| roe deer 16 III | GU236882 | 3 |
| roe deer 19 | GU236884 | 3 |
| roe deer 1972 39 | GU236898 | 2 |
| roe deer 2 | GU236865 | 2 |
| roe deer 22 | GU236887 | 3 |
| roe deer 25 | GU236890 | 2 |
| roe deer 26 | GU236891 | 3 |
| roe deer 27 | GU236892 | 2 |
| roe deer 28 | GU236893 | 3 |
| roe deer 3 | GU236866 | 3 |
| roe deer 4 | GU236867 | 2 |
| roe deer 470 | GU236903 | 2 |
| roe deer 5 | GU236868 | 2 |
| roe deer 794 | GU236907 | 2 |
| roe deer 8 | GU236871 | 3 |
| roe deer 9 | GU236872 | 2 |
| sheep 26 | GU236751 | 4 |
| sheep 31 | GU236753 | 4 |
| sheep 39 | GU236754 | 4 |
| sheep 5004NSV | GU236780 | 4 |
| sheep 5006EM | GU236756 | 4 |
| sheep 5009EM | GU236757 | 1 |
| sheep 5011EM | GU236759 | 1 |
| sheep 5041NSV | GU236786 | 4 |
| sheep 5051NSV | GU236787 | 4 |
| sheep 5064EM | GU236762 | 4 |
| sheep 5081EM | GU236765 | 4 |
| sheep 5093TM | GU236771 | 1 |
| sheep 5623TM | GU236776 | 4 |
| sheep 5626TM | GU236777 | 4 |
| sheep 7 | GU236748 | 4 |
| sheep 8 | GU236749 | 4 |
| sheep adult1KK | GU236755 | 1 |
| sheep SH13 | GU236795 | 1 |
| sheep SH16 | GU236798 | 4 |
| sheep SH5 | GU236790 | 4 |
| sheep SH6 | GU236791 | 1 |
| sheep variant1 | GQ428334 | 4 |
| shrew S1 | KC740475 | 5 |
| tick 84689 | MN062926 | This study |
| tick D12 | KF242723 | 2 |
| tick D13 | KF242724 | 4 |
| tick D20 | AY282368 | 2 |
| tick G35 | AY282372 | 1 |
| tick I121 | AY282374 | 1 |
| tick I20 | AY282375 | 2 |
| tick I68 | AY282377 | 1 |
| tick I94 | KF242730 | 4 |
| tick K71 | AY282378 | 1 |
| tick W37 | AY282388 | 1 |
| wild boar 10123 | JQ347563 | 1 |
